# Atrial Function in Patients with Breast Cancer After Treatment with Anthracyclines

**DOI:** 10.5935/abc.20160146

**Published:** 2016-11

**Authors:** Yalin Tolga Yaylali, Ahmet Saricopur, Mustafa Yurtdas, Hande Senol, Gamze Gokoz-Dogu

**Affiliations:** 1 Pamukkale University Faculty of Medicine, Cardiology Denizli; - Turkey; 2 Balikesir Sevgi Hospital; - Turkey; 3 Pamukkale University Dept of Biostatistics; - Turkey; 4 Pamukkale University Medical Oncology - Turkey

**Keywords:** Atrial Function, Arrhythmias, Cardiac, Unilateral Breast Neoplasms, Antrhacyclines, Drug Therapy, Cardiotoxicity

## Abstract

**Background:**

Atrial electromechanical delay (EMD) is used to predict atrial fibrillation, measured by echocardiography.

**Objectives:**

The aim of this study was to assess atrial EMD and mechanical function after anthracycline-containing chemotherapy.

**Methods:**

Fifty-three patients with breast cancer (48 ± 8 years old) who received 240 mg/m^2^of Adriamycin, 2400 mg/m^2^ of cyclophosphamide, and 960 mg/m^2^ of paclitaxel were included in this retrospective study, as were 42 healthy subjects (47 ± 9 years old). Echocardiographic measurements were performed 11 ± 7 months (median 9 months) after treatment with anthracyclines.

**Results:**

Left intra-atrial EMD (11.4 ± 6.0 vs. 8.1 ± 4.9, p=0.008) and inter-atrial EMD (19.7 ± 7.4 vs. 14.7 ± 6.5, p=0.001) were prolonged; LA passive emptying volume and fraction were decreased (p=0.0001 and p=0.0001); LA active emptying volume and fraction were increased (p=0.0001 and p=0.0001); Mitral A velocity (0.8 ± 0.2 vs. 0.6 ± 0.2, p=0.0001) and mitral E-wave deceleration time (201.2 ± 35.6 vs. 163.7 ± 21.8, p=0.0001) were increased; Mitral E/A ratio (1.0 ± 0.3 vs. 1.3 ± 0.3, p=0.0001) and mitral Em (0.09 ± 0.03 vs. 0.11 ± 0.03, p=0.001) were decreased; Mitral Am (0.11 ± 0.02 vs. 0.09 ± 0.02, p=0.0001) and mitral E/Em ratio (8.8 ± 3.2 vs. 7.6 ± 2.6, p=0.017) were increased in the patients.

**Conclusions:**

In patients with breast cancer after anthracycline therapy: Left intra-atrial, inter-atrial electromechanical intervals were prolonged. Diastolic function was impaired. Impaired left ventricular relaxation and left atrial electrical conduction could be contributing to the development of atrial arrhythmias.

## Introduction

The anthracyclines (AC), which are a key component of many chemotherapy regimens, are clearly the most cardiotoxic chemotherapeutic agents producing left ventricular dysfunction and arrhythmias.^1^ Atrial conduction system abnormalities play a major role in the genesis of re-entrant atrial arrhythmias. Left atrial (LA) volume and mechanical function have recently been identified as a potential indicator of cardiac dysfunction and arrhythmias.^2,3^ Intra- and inter-atrial electromechanical delays are well-known electrophysiological features of the atrium prone to fibrillation.^4^ Echocardiography is a sensitive and reproducible technique for the assessment of atrial mechanical and electromechanical features.^5,6^ Atrial electromechanical delay had been shown to be prolonged in patients with nonrheumatic paroxysmal atrial fibrillation, obesity, hyperthyroidism, and celiac disease.^7-10^ The pathophysiology and predictors of arrhythmias after treatment with AC are poorly defined. The hypothesis of the present study was that atrial mechanical and electromechanical features might be affected in patients treated with AC. Therefore, we aimed to assess atrial mechanical and electromechanical features in patients after the administration of anthracycline chemotherapy and to compare them with healthy controls and then to evaluate their relationship with parameters of left ventricular function on echocardiography.

## Methods

### Study population

The study design was retrospective. Fifty-three women with breast cancer were selected from consecutive patients who received 240 mg/m^2^ of Adriamycin, 2400 mg/m^2^ of cyclophosphamide, and 960 mg/m^2^ of paclitaxel at our institution between January 1, 2013 and December 31, 2013. A power analysis was performed before the study. Accordingly, when a reference study was considered, 28 subjects (14 in each group) would have resulted in 95% confidence and 90% power. We included 95 subjects (53 patients and 42 controls) in the present study. The power analysis showed that our results, when examined for inter-atrial EMD values, reached 95% confidence and 94% power. These women had a comprehensive echocardiographic examination before the treatment, which showed truly normal LV systolic and diastolic function. The control group was comprised by 42 age-and sex-matched healthy female office staff. Exclusion criteria were ischemic heart disease, moderate-to-severe valvular heart disease, heart failure, hypertension, diabetes mellitus, obesity, systolic and/or diastolic dysfunction, atrial fibrillation, bundle branch block, atrioventricular conduction abnormalities on electrocardiogram, acute or chronic renal failure, collagen tissue disease, thyroid dysfunction, electrolyte imbalance, pulmonary disease, anemia, chronic liver disease, prior history of radiation therapy, life expectancy< 1 year, and insufficient echocardiographic imaging. None of the participants were taking any antiarrhythmics, tricyclic antidepressants, antihistaminics, angiotensin-converting enzyme inhibitors, angiotensin receptor blockers, and antipsychotics. Heart rate, blood pressure, and routine biochemistry were measured in all participants. Exercise tolerance test to exclude ischemic heart disease was performed in all subjects. A total of 750 women with breast cancer were examined as to their eligibility for our study.

The institution's Medical Ethics Review Committee approved the study protocol (registration number: 60116787-020/7763, date of issue: 06.02.2014). Informed consent was obtained from all participants. The study protocol conforms to the ethical guidelines of the 1975 Declaration of Helsinki.

### Transthoracic echocardiography

**Figure 1:**
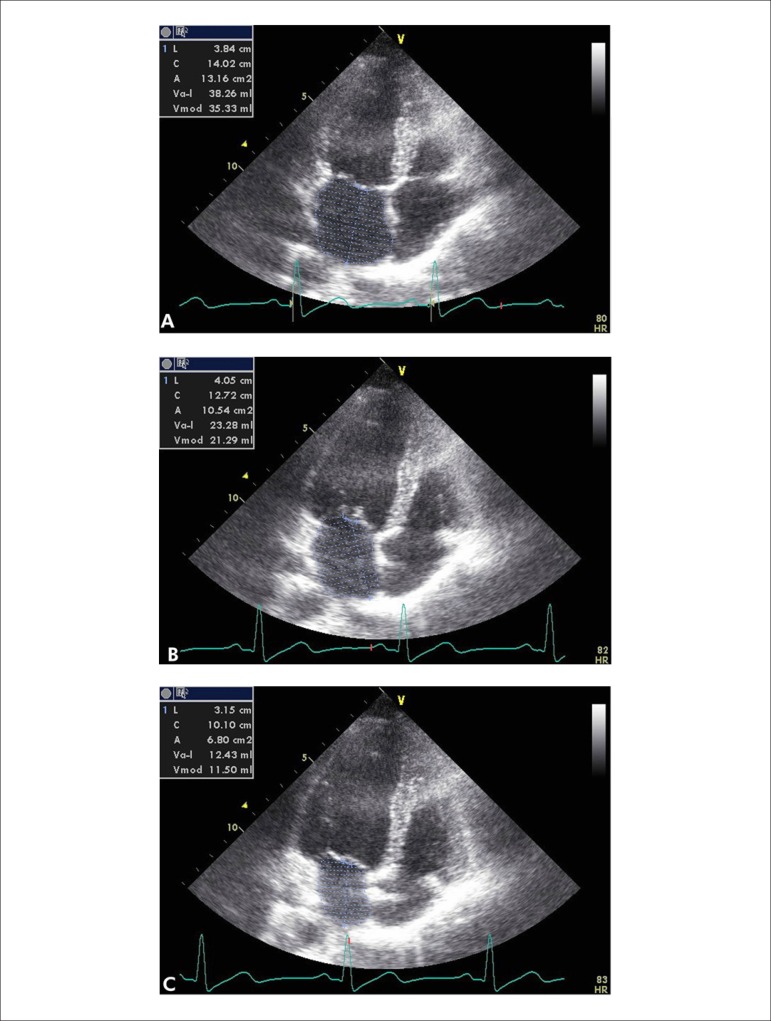
LA volumes are measured in the A4C views by means of 2D Echo at the mitral valve opening (A), at the onset of atrial systole (B), and at the mitral valve closure (C).

**Figure 2:**
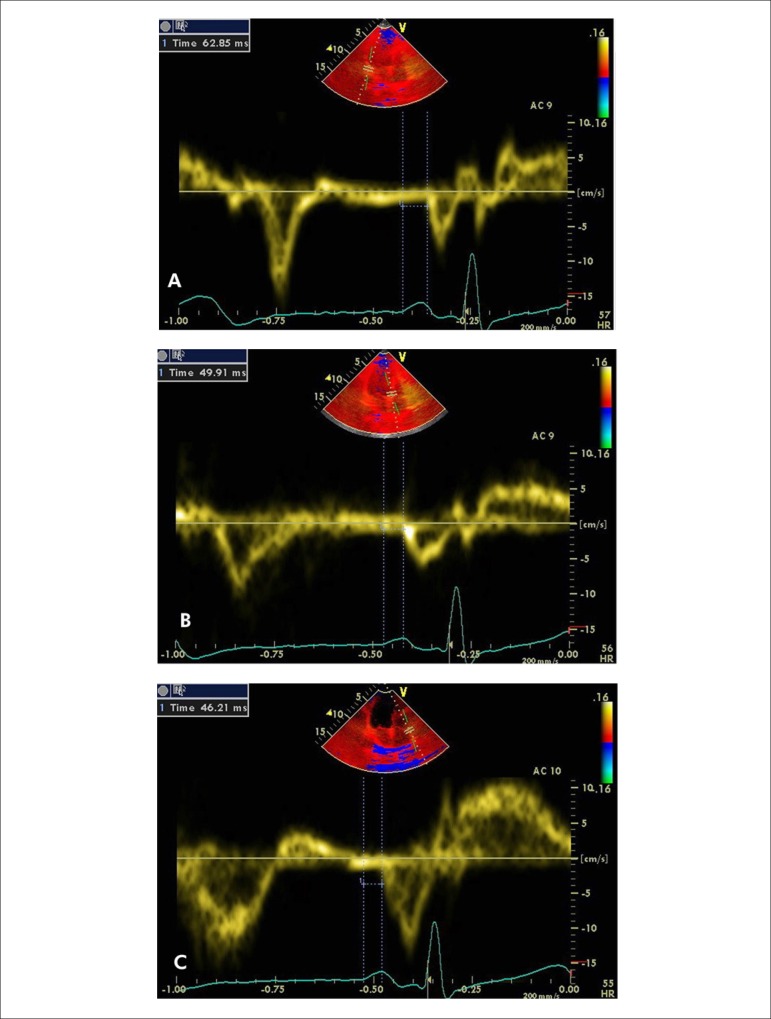
Measurements of time interval from the onset of P wave to the beginning of Am wave (PA) are obtained at the lateral mitral annulus (A), septal mitral annulus (B), and RV tricuspid annulus (C).

Echocardiographic examinations were carried out by using Vivid-7 echocardiography device with a 2.5-4 MHz probe (GE VingmedUltrasound, Horten, Norway). All participants were examined in the left lateral decubitus position by 2-D, M-mode, pulsed and color flow Doppler, and tissue Doppler echocardiography. Continuous 1-lead ECG recording was performed during the examination. LV ejection fraction was obtained from two- or apical four-chamber views through the modified Simpson method. To obtain maximum filling velocities, the pulsed-Doppler sampling volume was placed between the tips of the mitral valve leaflets in the apical four-chamberview. Myocardial velocity profiles of the lateral and septal mitral annuli were obtained. Tissue Doppler measurements were obtained by placing the sample volume at the junction of the mitral annulus and the septum, and lateral wall. All measurements were recorded as an average of 3 cardiac cycles. Left atrial volumes (LA V) were obtained from apical four-chamber view through the discs method and indexed for body surface area (BSA). LA V measurements were performed at the mitral valve opening (maximal, Vmax), the onset of atrial systole (P wave on electrocardiogram, Vp), and the mitral valve closure (minimal, Vmin) (Figure 1 A-C). The following LA emptying function parameters were calculated: LA passive emptying volume = Vmax-Vp, LA passive emptying fraction = (Vmax-Vp)/Vmax, LA active emptying volume = Vp-Vmin, LA active emptying fraction = (Vp-Vmin)/Vp.^10^ All volumes were indexed to BSA and expressed in ml/m^2^. Atrial electromechanical coupling (PA) was defined as the time interval from the onset of the P wave on surface electrocardiogram to the beginning of the late diastolic wave (Am wave). PA was obtained from the lateral mitral annulus (PA lateral), septal mitral annulus (PA septum), and right ventricular tricuspid annulus (PA tricuspid) (Figure 2 A-C). Values were averaged over 3 consecutive beats. The difference between PA lateral and PA tricuspid was defined as inter-atrial electromechanical delay, the difference between PA lateral and PA septum was defined as left intra-atrial electromechanical delay, and the difference between PA septum and PA tricuspid was defined as right intra-atrial electromechanical delay.^10^

### Reproducibility

One experienced operator blinded to clinical and laboratory characteristics of the participants analyzed all echocardiographic data. Intraobserver variability was assessed in 30 selected subjects randomly from the both groups by repeating the measurements. Measurements were repeated 1 week apart. Only one reader conducted this analysis. The reader was allowed to select the best measurement each time and was blinded to previous measurements. Intraobserver variability was calculated as the difference between two measurements of the same patient by a single cardiologist divided by the mean value.

### Statistical analysis

Categorical variables are expressed as percentages and continuous variables as mean ± standard deviation and median with minimum-maximum values. Continuous variables were compared between groups using independent t tests (for normally distributed variables) or a Mann-Whitney U test (for variables not normally distributed). Categorical data were compared with the chi-square test. A multiple linear regression analysis was used to identify independent predictors of LA mechanical function impairment and electromechanical delays. P values < 0.05 were considered to indicate statistical significance. All analyses were performed using SPSS version 15.0 (SPSS, Inc., Chicago, IL).

## Results

Clinical and echocardiographic findings of the patients (53 women, 48 ± 8 years) and controls (42 women, 47 ± 9 years) are listed in Table 1. None of the patients had developed cardiac complications including atrial fibrillation. Age, weight, body mass index, body surface area, systolic and diastolic blood pressures, heart rate, and LV EF were similar between the 2 groups. The mean duration of time elapsed from the completion of chemotherapy to the performance of echocardiography was 11 ± 7 months (median 9 months). The mitral A-wave velocity, E-wave deceleration time, Am velocities, E/Em ratio were significantly higher in the patients. The mitral Em velocities, E/A ratio were significantly lower in the patients. The other conventional echocardiographic parameters such as LV EF, IVS and PW thickness, LA diameter, LVEDD, LVESD, and systolic pulmonary artery values were within normal reference ranges, with no significant differences between the 2 groups (data not shown).

**Table 1:** Characteristics of the study subjects

	Patients (n=53)		Controls (n=42)		
	Mean ± SD	Med. (Min. – Max.)	Mean ± SD	Med. (Min. – Max.)	p Value
**Demographic And clinical characteristics**					
Age, years	48 ± 8	49(29 - 65)	46 ± 9	47(30 - 65)	0.419
Body Mass Index, kg/m^2^	23.98 ± 2.41	23.8 (19.5 – 28.1)	23.72 ± 2.33	23.8 (20.3 – 28.5)	0.603
Body Surface Area, m^2^	1.77 ± 0.14	1.8(1.5 – 2.1)	1.74 ± 0.13	1.7(1.5 – 2.1)	0.142
Systolic Blood Pressure, mmHg	118 ± 9	120 (100 – 135)	120 ± 9	120 (100 – 135)	0.396
Diastolic Blood Pressure, mmHg	71 ± 5	70 (60 – 85)	73 ± 5	73 (60 – 85)	0.064
Heart rate, beats/min	72 ±11	70 (64-90)	74 ± 11	72 (62-88)	0.104
LV Ejection Fraction, %	63 ± 5	63 (49 - 74)	64 ± 5	62(57-78)	0.958
**Effect of chemotherapy on Doppler parameters**					
Mitral E velocity, m/s	0.8 ± 0.2	0.8 (0.5-1.1)	0.8 ± 0.2	0.7 (0.4-1.2)	0.223
Mitral A velocity, m/s	0.8 ± 0.2	0.8 (0.5–1.2)	0.7 ± 0.2	0.6 (0.4-1.0)	0.0001*
Mitral E deceleration time, m/s	201.3 ± 35.6	196.3 (126-291.1)	163.7 ± 21.8	166.2 (109.9-203.7)	0.0001*
Mitral E/A ratio	1.0 ± 0.3	0.9 (0.5-1.9)	1.3 ± 0.3	1.3 (0.7-2.3)	0.0001*
Mitral Mean Em, m/s	0.09 ± 0.03	0.09 (0.05-0.16)	0.11 ± 0.03	0.11 (0.05-0.2)	0.001*
Mitral Mean Am, m/s	0.11 ± 0.02	0.11(0.06 – 0.14)	0.09 ± 0.02	0.09 (0.06-0.12)	0.0001*
Mitral E/Mean Em ratio	8.8 ± 3.2	8.7 (3.9-23.1)	7.6 ± 2.6	7.1 (4.1-16.8)	0.017*

* p<0,05 statistically significant; SD: standard deviation; Med: median; Min-Max: minimum - Maximum Values

### Left atrial mechanical functions

LA volume measurements of the study subjects are presented in Table 2. Maximum LA volume, Vp, LA active emptying volume (LAAEV) and fraction were significantly increased in the patients. LA passive emptying volume (LAPEV) and fraction were significantly decreased in the patients. The mitral E-wave deceleration time was weakly associated with LA passive emptying fraction (LAPEF), LA active emptying volume, and fraction. The reservoir function and total LA emptying did not change in the patients (data not shown).

**Table 2:** Left atrial volume measurements of the study subjects

	Patients (n=53)		Controls (n=42)		
	Mean ± SD	Med. (Min. – Max.)	Mean ± SD	Med. (Min. – Max.)	p Value
Effect of chemotherapy on atrial function					
Vmax^1^, ml/m^2^	23.76 ± 5.2	22.82 (16.54 – 38.81)	20.71 ± 3.22	20.6 (14.71 – 28.61)	0.001*
Vp^2^, ml/m^2^	16.34 ± 4.55	15.65 (10.41 – 31.57)	11.07 ± 2.19	11.29 (6.27 – 14.88)	0.0001*
Vmin^3^, ml/m^2^	8.78 ± 2.98	8.33 (4.66 – 18.95)	7.7 ± 1.79	7.96 (4 – 10.31)	0.153
LA passive emptying volume, ml/m^2^	7.41 ± 2.01	7.42 (3.01 – 12.32)	9.64 ± 2	9.71(4.92 – 14.54)	0.0001*
LA passive emptying fraction, %	31.6 ± 7.37	31.02 (15.8 – 45.49)	46.53 ± 7.03	45.34 (31.33-67.51)	0.0001*
LA active emptying volume, ml/m^2^	7.56 ± 2.21	7.25 (4.66-16.04)	3.41 ± 0.74	3.45 (2.15-4.75)	0.0001*
LA active emptying fraction, %	46.67 ± 7.04	45.45 (33-63.19)	31.19 ± 5.24	31.66 (19.73-44.77)	0.0001*

* p<0,05 statistically significant; SD: standard deviation; Med: median; Min-Max: minimum - Maximum Values.

1Vmax: Volume measurements were performed at the mitral valve opening; 2Vp: Volume measurements were performed at the onset of atrial systole; 3Vmin: Volume measurements were performed at the mitral valve closure. LA: Left atrial.

### Atrial electromechanical coupling

The atrial electromechanical coupling intervals measured from different sites are shown in Table 3. PA lateral, inter-atrial, and left intra-atrial electromechanical delays were significantly higher in the patients. However, PA septum, PA tricuspid, and right intra-atrial electromechanical delays did not differ significantly between the groups. E/A ratio was weakly associated with left intra-atrial and inter-atrial electromechanical delays (data not shown).

**Table 3:** Atrial electromechanical coupling findings measured by tissue Doppler imaging

	Patients (n=53)		Controls (n=42)		p Value
	Mean ± SD	Med. (Min. – Max.)	Mean ± SD	Med. (Min. – Max.)	
Effect of chemotherapy on atrial function					
PA lateral, ms	59.12 ± 8.91	59.15 (36.97-78.82)	54.04 ± 9.43	51.76 (36.97-73.94)	0.008*
PA septum, ms	47.72 ± 9.1	48.06 (29.57-69.54)	45.91 ± 9.5	44.36 (25.88-66.85)	0.348
PA tricuspid, ms	39.18 ± 8.36	40.67 (22-57.3)	39.35 ± 8.71	37.9 (22.18-62.85)	0.921
Inter-atrial EMD, ms	19.73 ± 7.38	18.49 (8.06-38.82)	14.69 ± 6.51	13.7 (7.39-29.58)	0.001*
Left intra-atrial EMD, ms	11.4 ± 5.98	10.76 (1.85-25.88)	8.13 ± 4.87	7.39 (1.84-22.18)	0.008*
Right intra-atrial EMD, ms	8.52 ± 5.48	7.39 (1.85-25.18)	6.56 ± 4.09	4.77 (2.61 – 22.18)	0.194

* p<0,05 statistically significant; SD: Standard Deviation; Med: Median; Min-Max: Minimum - Maximum Values.

EMD: Electromechanical delay; PA: The time interval from the onset of P wave on surface electrocardiogram to the beginning of Am wave with TDI.

Among patients, the body mass index, diastolic blood pressure, mitral E deceleration time were independent predictors for LAPEF. The systolic blood pressure, diastolic blood pressure, mitral E deceleration time were independent predictors for LAAEV. Among controls, age, mitral E/A ratio were independent predictors for LAAEF (Table 4).

Table 4Multiple linear regression analysis of subject characteristics influencing LA mechanical function impairment

**Table:** 

Patient Group Dependent Variable: LAPEF	Standardized Beta	sig. (p)	95,0% CI Lower - Upper
Age	-0,098	0,505	-0,334 - 0,167
BMI	0,292	0,037*	0,054 - 1,733
SBP	-0,312	0,058	-0,491 - 0,008
DBP	0,575	0,001*	0,303 - 1,118
Mitral E/A ratio	-0,009	0,949	-6,964 - 6,53
Mitral E deceleration time	-0,271	0,039*	-0,109 - -0,003
Mitral E/Mean Em ratio	0,053	0,696	-0,507 - 0,752

R^2^=0,321; model p=0,01; BMI: body mass index; CI: confidence interval; DBP: diastolic blood pressure; LAPEF: left atrial passive emptying fraction; SBP: systolic blood pressure;

* statistically significant.

**Table:** 

Patient Group Dependent Variable: LAAEV	Standardized Beta	sig. (p)	95,0% CI Lower - Upper
Age	-0,042	0,783	-0,089 - 0,067
BMI	-0,186	0,196	-0,432 - 0,091
SBP	0,461	0,008*	0,029 - 0,184
DBP	-0,576	0,001*	-0,341 - -0,086
Mitral E/A ratio	-0,010	0,948	-2,17 - 2,034
Mitral E deceleration time	0,285	0,036*	0,001 - 0,034
Mitral E/Mean Em ratio	0,076	0,587	-0,143 - 0,249

R^2^=0,152; model p=0,041; BMI: body mass index; CI: Confidence Interval; DBP: diastolic blood pressure; LAAEV: left atrial active emptying volume; SBP: systolic blood pressure;

* statistically significant.

**Table:** 

Control Group Dependent Variable: LAPEF	Standardized Beta	sig. (p)	95,0% CI Lower - Upper
Age	-0,541	0,005*	-0,509 - -0,101
BMI	-0,311	0,065	-1,445 - 0,045
SBP	0,189	0,213	-0,065 - 0,283
DBP	-0,100	0,508	-0,359 - 0,181
Mitral E/A ratio	-0,437	0,017*	-12,325 - -1,293
Mitral E deceleration time	-0,103	0,493	-0,098 - 0,048
Mitral E/Mean Em ratio	0,221	0,168	-0,199 - 1,1

R^2^=0,243; model p=0,018; BMI: body mass index; CI: Confidence Interval; DBP: diastolic blood pressure; LAPEF: left atrial passive emptying fraction; SBP: systolic blood pressure;

* statistically significant.

Intra-observer variability was less than 5% for all echocardiographic measurements.

## Discussion

The present study showed that left intra-atrial and inter-atrial EM intervals were prolonged; LV diastolic function was impaired in patients with breast cancer treated with AC, despite preservation of LA mechanical functions.

This study suggests that proarrhythmogenic mechanisms could be the result of changes in cardiac function, including LV diastolic dysfunction and delayed electrical conduction, which create an arrhythmogenic substrate. This arrhythmogenic substrate leads to a decrease in intracardiac conduction and to a heterogeneous dispersion of repolarization, two effects that facilitate the genesis of cardiac arrhythmias in those patients. This study also showed that diastolic function parameters were significantly impaired in the patients. We were not able to demonstrate systolic dysfunction in our patients because we could not perform global strain evaluation. The most common cardiotoxicity of AC is left ventricular systolic dysfunction with possible arrhythmias. Studies examining the occurrence of arrhythmia in patients treated with AC are scarce. Atrial fibrillation appears to be a rather common complication of AC and in one study, it was described in 2-10% of the patients.^11^ There are a limited number of studies investigating diastolic function in patients treated with AC. There is no consensus regarding whether diastolic function is impaired in those patients.

In this study, patients with breast cancer after AC did not display any significant change in total LA emptying, despite the fact that we showed that LA active emptying volume and LA active emptying fraction were increased in those patients. This may suggest preservation of atrial mechanical function early in the course of breast cancer survivors treated with AC. Yet it is possible to consider that the decrease in LA passive emptying volume is related to elevated end-diastolic LV pressure at least due to LV diastolic dysfunction. The shift in early and late diastolic ventricular filling might have occurred secondary to impaired relaxation. LA mechanical function plays a significant role to maintain cardiac output in patients with DD.^12^ LA mechanical function is an important determinant of LV filling, especially in patients with end-stage systolic or diastolic ventricular dysfunction.^12^ It consists of reservoir, conduit, and booster pump functions. In previous studies, it was demonstrated that LA mechanical functions were impaired in patients with hypertension, chronic obstructive lung disease, and type 1 diabetes mellitus.^4,13,14^ In addition, LA volume has been shown to be a powerful prognostic variable in a variety of cardiac disease states. Compared with AP diameter, LA volume has a stronger association with outcomes in cardiac patients.^15^ Left atrial total emptying fraction was reported to be an independent and strong predictor for the development of AF.^10^

The present study revealed that left intra- and interatrial EMD were significantly prolonged in the patients. Only mitral E/A ratio was weakly associated with LA EMD. Increased EM delay was found to assist in the identification of subjects at increased risk of AF. Atrial conduction time reflects both electrical and structural atrial remodeling. Atrial EMD has been reported to be associated with low-grade inflammation, insulin resistance, LA enlargement, early LV diastolic dysfunction, and oxidative stress.^4,16-18^ Therefore, the impaired conduction observed in the present study could be associated with an increased risk of AF.

The prolonged EM intervals observed in this study could be explained by anthracycline-induced oxidative stress, which has been associated with autonomic dysfunction.^18,19^ Prolonged EM intervals may also indicate atrial remodeling during arrythmogenic process and may be a predictor of AF.^7,20^ Autonomic nervous system may play a role in the development of atrial fibrillation by its effects on atrial conduction time (heterogeneity).^21,22^ Studies looking at atrial function in patients treated with cardiotoxic chemotherapy are limited. Our results are consistent with a previous study by Ceyhan et al., which reported that intra- and inter-atrial conductions were delayed in patients treated with 5-florouracil.^23^

The results of our multiple linear regression analysis are inconclusive due to low R² values. They may suggest that risk factors for diastolic dysfunction such as blood pressure, BMI, age, and diastolic dysfunction itself may play role in LA mechanical function impairment.

### Clinical implications

These alterations may be an early form of subclinical cardiac involvement in breast cancer survivors treated with AC and with no cardiovascular risk factors. We can speculate that these patients may be at a higher risk to develop new or recurrent atrial arrhythmias particularly atrial fibrillation. Monitoring could be prudent in those patients to guide additional interventions such as anti-arrhythmic and/or anti-coagulant therapy. This could lead to a better clinical management and improved patient outcome.

### Study limitations

In this study, patients also received cyclophosphamide and paclitaxel simultaneously, which makes it difficult to decide which one caused these adverse effects. Pretreatment evaluation with comprehensive echocardiography including assessment of atrial mechanical and electromechanical features had not been done. However, the patients were selected among the ones who had truly normal LV systolic and diastolic function on the baseline studies. These alterations might have been attributed incorrectly to the adverse effects of chemotherapeutic agents. LA volume should be measured using the disk summation algorithm in both the apical four- and two- chamber views. We had measured them from a single-plane apical four-chamber approach, which is typically 1 to 2 mL/m^2^ smaller than apical two-chamber volumes.^15^ However, we used the same technique for both patients and controls. We excluded patients with cardiovascular risk factors that can create an arrhythmogenic substrate. We cannot rule out systolic dysfunction, as we could not study global strain. Further research is needed to define a true incidence and clinical relevance of atrial arrhythmias in those patients and to determine the role of Holter monitoring for the early diagnosis, intervention and surveillance of those patients more susceptible to develop arrhythmia.

## Conclusions

Our study revealed that left intra- and inter-atrial EM were prolonged and that LV diastolic function was impaired in breast cancer survivors treated with AC. Impaired electrical conduction in those patients may be associated with the development of arrhythmias.

## References

[1] Chen MH, Force T, Mann DL, Zipes DP, Libby P, Bonow RO (2015). Cardiovascular complications of cancer therapeutic
agents. Braunwald's heart disease: a textbook of cardiovscular medicine.

[2] Abecasis J, Dourado R, Ferreira A, Saraiva C, Cavaco D, Santos KR (2009). Left atrial volume calculated by multi-detector computed
tomography may predict successful pulmonary vein isolation in catheter
ablation of atrial fibrillation. Europace.

[3] Hof I, Chilukuri K, Arbab-Zadeh A, Scherr D, Dalal D, Nazarian S (2009). Does left atrial volume e Pulmonary venous anatomy predict the
outcome of catheter ablation of atrial fibrillation?. J Cardiovasc Electrophysiol.

[4] Acar G, Akcay A, Sokmen A, Ozkaya M, Guler E, Sokmen G (2009). Assessment of atrial electromechanical delay, diastolic
functions, and left atrial mechanical functions in patients with type 1
diabetes mellitus. J Am Soc Echocardiogr.

[5] Deniz A, Yavuz B, Aytemir K, Hayran M, Kose S, Okutucu S (2009). Intra-left atrial mechanical delay detected by tissue Doppler
echocardiography can be a useful marker for paroxysmal atrial
fibrillation. Echocardiography.

[6] Omi W, Nagai H, Takamura M, Okura S, Okajima M, Furusho H (2005). Doppler tissue analysis of atrial electromechanical coupling in
paroxysmal atrial fibrillation. J Am Soc Echocardiogr.

[7] Cui QQ, Zhang W, Wang H, Sun X, Wang R, Yang HY (2008). Assessment of atrial electromechanical coupling and influential
factors in nonrheumatic paroxysmal atrial fibrillation. Clin Cardiol.

[8] Yagmur J, Cansel M, Acikgoz N, Ermis N, Yagmur M, Atas H (2011). Assessment of atrial electromechanical delay by tissue Doppler
echocardiography in obese subjects. Obesity (Silver Spring).

[9] Sokmen A, Acar G, Sokmen G, Akcay A, Akkoyun M, Koroglu S (2013). Evaluation of atrial electromechanical delay and diastolic
functions in patients with hyperthyroidism. Echocardiography.

[10] Bayar N, Çekin AH, Arslan Ş, Çağırcı G, Erkal Z, Çay S (2015). Assessment of left atrial function in patients with celiac
disease. Echocardiography.

[11] Guglin M, Aljayeh M, Saiyad S, Ali R, Curtis AB (2009). Introducing a new entity: chemotherapy-induced
arrhythmia. Europace.

[12] Prioli A, Marino P, Lanzoni L, Zardini P (1998). Increasing degrees of left ventricular filling impairment
modulate left atrial function in humans. Am J Cardiol.

[13] Aydin M, Ozeren A, Bilge M, Dursun A, Cam F, Elbey MA (2004). Effects of dipper and non-dipper status of essential hypertension
on left atrial mechanical functions. Int J Cardiol.

[14] Acikel M, Yilmaz M, Gurlertop Y, Kaynar H, Bozkurt E, Erol MK (2004). The effect of pulmonary hypertension on left atrial mechanical
functions in chronic obstructive lung disease. Int J Cardiol.

[15] Lang RM, Badano LP, Mor-Avi V, Afilalo J, Armstrong A, Ernande L (2015). Recommendations for cardiac chamber quantification by
echocardiography in adults: an update from the American Society of
Echocardiography and the European Association of Cardiovascular
Imaging. J Am Soc Echocardiogr.

[16] Zehir R, Karabay CY, Kocabay G, Kalayci A, Kaymaz O, Aykan AC (2014). Assessment of atrial conduction time in patients with polycystic
ovary syndrome. J Interv Card Electrophysiol.

[17] Yagmur J, Yetkin O, Cansel M, Acikgoz N, Ermis N, Karakus Y (2012). Assessment of atrial electromechanical delay and influential
factors in patients with obstructive sleep apnea. Sleep Breath.

[18] Acar G, Sayarlioğlu M, Akçay A, Sökmen A, Sökmen G, Yalçintaş S (2009). Evaluation of atrial electromechanical delay and left atrial
mechanical functions in patients with rheumatoid arthritis. Turk Kardiyol Dern Ars.

[19] Minotti G, Cairo G, Monti E (1999). Role of iron in antraciclina cardiotoxicity: new tunes for an old
song?. FASEB J.

[20] Aktoz M, Yilmaztepe M, Tatli E, Turan FN, Umit EG, Altun A (2011). Assessment of ventricular and left atrial mechanical functions,
atrial electromechanical delay e P wave dispersion in patients with
scleroderma. Cardiol J.

[21] Roshanali F, Mandegar MH, Yousefnia MA, Alaeddini F, Saidi B (2009). Prevention of atrial fibrillation after coronary artery bypass
grafting via atrial electromechanical interval and use of amiodarone
prophylaxis. Interact Cardiovasc Thorac Surg.

[22] Workman AJ (2010). Cardiac adrenergic control and atrial
fibrillation. Naunyn Schmiedebergs Arch Pharmacol.

[23] Ceyhan C, Meydan N, Barutca S, Tekten T, Onbasili AO, Ozturk B (2005). Ultrasound tissue characterization by integrated backscatter for
analyzing Fluorouracil induced myocardial damage. Echocardiography.

